# Loneliness and socioemotional memory

**DOI:** 10.1111/bjso.12783

**Published:** 2024-06-29

**Authors:** Tasuku Igarashi

**Affiliations:** ^1^ Graduate School of Education and Human Development Nagoya University Nagoya Japan

**Keywords:** autobiographical memory, emotion, linguistic inquiry and word count, loneliness

## Abstract

Do chronically high‐lonely individuals exhibit specific memory biases when recalling past social episodes? We explored negative memory biases, focusing on the recall of unfavourable social experiences and social memory biases, emphasizing the recall of social experiences irrespective of emotional valence. We conducted a dictionary‐based semantic analysis of autobiographical episodes obtained from 4095 participants via four datasets. Participants recalled a positive, negative or emotionally neutral episode from their recent past. High‐lonely individuals predominantly exhibited a decline in recalling positive social episodes, providing partial support for negative memory biases. However, both high‐ and low‐lonely individuals were similarly inclined to recall negative social episodes. These results suggest that the primary issue among high‐lonely individuals is the limited recall of positive social experiences rather than the general negativity in memory biases or the hypersensitivity to general social memories.

## INTRODUCTION

Loneliness is a largely unwanted and unchosen experience. Although a socially flexible environment, characterized by freedom in selecting relationships, offers substantial advantages to both individuals and society, it can also lead to individuals struggling with loneliness (Luhmann et al., 2023). The World Health Organization Commission on Social Connection has declared that loneliness, being both widespread and distressing, poses serious public health risks (World Health Organization, 2023). Loneliness is defined as a subjective negative emotional state of social dissatisfaction (Russell et al., 1978), representing a complex psychological condition with negative outcomes. Loneliness can lead to adverse physiological and psychological consequences such as short‐term declines in immune function and cognitive performance as well as long‐term risks of cardiovascular disease and early morbidity (Cacioppo & Patrick, 2008).

One possible mechanism by which loneliness impairs physical health is shifts in cognition towards social information. Loneliness signals a survival risk associated with the absence of social ties, prompting cognitive shifts towards heightened awareness and processing of social information as a protective mechanism (Cacioppo & Cacioppo, 2018). Cognitive shifts resulting from loneliness could intensify stress, weaken immune capabilities and increase the likelihood of negative health outcomes (Quadt et al., 2020). Understanding the mechanism of cognitive shifts is key to comprehending the profound impact of loneliness on psychosocial well‐being.

### Reaffiliation model and regulatory loop model of loneliness

The reaffiliation model of loneliness (Qualter et al., 2015) posits that the discomfort of loneliness initially prompts avoidance from social contexts, but the perception of isolation concurrently activates a cognitive reaffiliation process that makes individuals more sensitive to social cues to approach others. Hypervigilance towards social information occurs through two distinct pathways (Qualter et al., 2015). On the one hand, individuals can regain acceptance from others and reduce loneliness. This process is evidenced by increased proficiency in detecting social cues such as sad and fearful faces (Vanhalst et al., 2017) and recognizing the faces of members within the group (Van Bavel et al., 2012) among high‐lonely individuals. This enhanced social sensitivity, termed the social monitoring system (Pickett et al., 2005), initially serves to protect against further isolation.

On the other hand, the adaptive mechanism may backfire if loneliness persists. Chronic loneliness can lead to a maladaptive pattern of social cognition, where individuals are more likely to perceive others negatively and avoid social interactions, thereby increasing their isolation (Spithoven et al., 2017). The regulatory loop model of loneliness (Cacioppo & Hawkley, 2009) posits that loneliness triggers a self‐perpetuating cycle of negative expectations about social interactions, leading to increased vigilance towards social threats and withdrawal. Consequently, loneliness further accelerates and completes a feedback loop that hinders the formation of positive social connections, perpetuating isolation. This paradoxical perspective highlights the complex nature of the impact of loneliness on cognition and social behaviour, illustrating how an initially adaptive response can become maladaptive over time (Qualter et al., 2015).

### Loneliness and autobiographical memory

In this study, we aim to investigate the impact of chronic loneliness on cognitive functions, particularly focusing on autobiographical memory. Memory is an essential cognitive function in human mental processes. The ‘cognitive aspects of loneliness’ model (Spithoven et al., 2017) emphasizes how loneliness is linked with cognitive biases across all stages of information processing, suggesting that negative social biases loom over high‐lonely individuals. These biases, particularly in memory function, play a central role in the emergence of further negative cognitive biases and may lead to deficits in social skills among high‐lonely individuals. Thus, loneliness not only propels the processing of social information but also drives the processing of negative information, impacting how individuals remember and recall their social environment (also see Yang, 2019 for a review).

Despite its importance, memory in loneliness has been largely unexplored. Loneliness is associated with episodic memory that relates to the recall of personal experiences; individuals high in loneliness tend to perform poorly on delayed recall tasks for verbal episodic memory (Luchetti et al., 2022). Although most existing studies focus on the relationship between the level of loneliness and memory task performance (Kang & Oremus, 2023), retrieving a list of specific words in a standardized cognitive task can lead to the loss of diverse details. In other words, the focus on quantitative metrics in memory often overlooks the qualitative aspects of what types of events, experiences and episodes are recalled. Examining the semantic aspects of episodic memory such as the social and personal nature of recalled emotional events can better explain how loneliness influences not only the quantity but also the content and tone of remembered experiences. This approach would further inform the development of targeted interventions (Nourkova & Vasilenko, 2018) to mitigate the adverse effects of cognitive functions on loneliness. Scrutinizing the connection between the processing of socioemotional information within autobiographical memory and the experience of loneliness offers valuable insights into the ramifications of loneliness on long‐term health.

Autobiographical memory is an integrated form of self‐related past experiences with social and emotional components, and is crucial for sociocultural adaptation and future planning (Conway & Pleydell‐Pearce, 2000; Harris et al., 2014; Nelson & Fivush, 2004). Autobiographical memory encompasses a temporal extension of events and lays the groundwork for the diverse and meaningful interpretation of the self and social life based on personal history (Fivush, 2011). Despite its importance, autobiographical memory has been primarily examined for its functional utility to loneliness, such as nostalgia (Zhou et al., 2008); the relationships between its components and chronic loneliness have not been adequately explored.

Examining autobiographical memory can shed light on the impact of chronic loneliness on the interpretation and recollection of social experiences. Autobiographical memory, with its dynamic and multifaceted components emerging through narrative structure and content, reveals deeply personal and subjective experiences containing self‐relevant knowledge at different levels of specificity that quantitative methods might overlook. Social interactions and activities often feature prominently in these memories (Fivush, 2011). Analysing autobiographical memory can thus provide valuable information about the quality and nature of social relationships, helping identify patterns of social interaction that contribute to feelings of loneliness.

### Negative memory biases and social memory biases

The core component of autobiographical memory includes emotion. In particular, negative memories are robust and easily accessed, with their durability stemming from the strength of encoding and integrating information (Williamson et al., 2019). Previous literature provides a prediction involving ‘negative memory biases’ for the association between chronic loneliness and socioemotional memory. High‐lonely individuals generally feel more negative and less positive emotions than low‐lonely individuals (Cacioppo et al., 2006) and prioritize processing negative social information (Cacioppo et al., 2009). These patterns are considered a result of the regulatory loop of loneliness. Therefore, it is possible that high‐lonely individuals recall more negative and fewer positive social episodes in autobiographical memory than low‐lonely individuals.

Meanwhile, research has shown that chronic loneliness is associated with increased recall of social information in general. This involves ‘social memory biases’, which represent a general tendency of high‐lonely individuals to recall social cues regardless of their emotional valence. Gardner et al. (2000) revealed that immediate experiences of rejection promoted the retention of social events over non‐social ones. In Gardner et al.'s (2005) social memory task, individuals high in chronic loneliness displayed heightened social attention and enhanced memory for both positive and negative social cues in another person's diary. These biases may reflect the hypersensitivity and expectations of high‐lonely individuals towards social engagements as a mixture of immediate/adaptive and chronic/maladaptive strategies to mitigate their social discomfort. Drawn from this empirical evidence, it is predicted that high‐lonely individuals recall both positive and negative social episodes in autobiographical memory.

### Linguistic expressions of social experiences

When recalling autobiographical memory, high‐lonely individuals may recall fewer positive social experiences related to others, reflecting the relative scarcity of such events and the increase in individual events in daily lives. While memory biases triggered by loneliness can occur regardless of the frequency of social interactions, loneliness is often correlated positively with negative social experiences (Katz et al., 2024) and negatively with network size (Igarashi, 2002), frequent social contacts (Kuczynski et al., 2022) and positive social experiences (Katz et al., 2024). Research using natural language processing to analyse millions of Twitter and Facebook posts (Guntuku et al., 2019; Liu et al., 2022) found that loneliness is associated with the use of fewer social words. Semantic analysis of a large collection of episodic memories regarding loneliness among Japanese participants revealed a similar finding (Iwai & Kumada, 2023). In parallel, negativity bias in general is associated with depression and the suppression of recalling positive events in one's life (Gotlib & Joormann, 2010). Including life events and depression as control variables in analysis is crucial to explore biases rooted in loneliness and autobiographical memory.

The hypersensitivity to specific socioemotional cues among high‐lonely individuals manifests in real‐world behaviours through linguistic expressions. Autobiographical memories encapsulate intricate ecological cues and provide a multifaceted perspective of one's psychological state across various dimensions. The words individuals use to describe their past experiences can provide insights into their underlying emotions and thoughts. Recently, language analysis has underscored the profound significance of verbal expressions in diverse contexts (Jackson et al., 2022; Tausczik & Pennebaker, 2010). This approach highlights the interconnectedness of language and psychological processes, facilitating a more profound understanding of human behaviour in its organic forms. For example, one study (Himmelstein et al., 2018) analysed the autobiographical memories of adults with major depressive disorder using the Linguistic Inquiry and Word Count (LIWC) (Pennebaker et al., 2015), a *de facto* standard platform for dictionary‐based text analysis, and found distinct linguistic patterns linked to depression severity such as the frequent use of a first‐person singular pronoun in negative contexts. However, existing literature has not focused on the social aspects of language, specifically the use of social words (i.e. words related to social activities such as communication, family and friends; Pressman & Cohen, 2007), while exploring the manipulation of emotional valence in autobiographical memory.

### Aims of the research

In the present study, we examine the associations between loneliness and the use of social words in autobiographical memories by manipulating the emotional valence of recalled episodes. If high‐lonely individuals exhibit negative memory biases, their autobiographical memory would contain fewer social episodes involving positive valence and more social episodes involving negative valence. If lonely individuals display general social memory biases, their memory would contain more social episodes regardless of emotional valence; additionally, high‐lonely individuals would naturally use more social words than low‐lonely individuals while describing episodes without specific emotional valence, such as ordinary daily episodes, in the absence of an explicit prompt to think about social interactions. This study contributes to methodological advancement and provides empirical evidence that challenges or substantiates existing theories on the cognitive aspects of loneliness.

## METHOD

We report all manipulations, measures and exclusions in these data. All data, scripts, instructions and materials (except the raw data of Dataset 1 collected by Igarashi, 2019) are publicly available at https://osf.io/s7qrg/.

### Data

We considered four datasets to examine the association between loneliness and social word use. Dataset 1 (Lancers2018) comprised existing text data reported in previous research (Igarashi, 2019; Igarashi et al., 2022), including responses from 522 Japanese crowdsourcing workers recruited from Lancers (a data collection platform) in June 2018.^1^ Datasets 2, 3 and 4 were collected for this study.^2^ Dataset 2 (Lancers2023) included responses from 563 Japanese crowdsourcing workers recruited from Lancers in July 2023. Dataset 3 (Yahoo2023) included responses from 1647 Japanese crowdsourcing workers recruited from Yahoo! Crowdsourcing in September 2023. Dataset 4 (Yahoo2024) included responses from 1671 Japanese crowdsourcing workers recruited from Yahoo! Crowdsourcing in February 2024. Dataset 1 was used for the initial confirmation of the association between loneliness and social word use; Dataset 2 was used to replicate findings from Dataset 1 on the same platform but in a different period; Dataset 3 was used to replicate the findings from Datasets 1 and 2 on a different platform with preregistration of the research design and analysis plans; and Dataset 4 was used to estimate the model including the control variables.

#### Sample size justification

We did not conduct an *a priori* power analysis for Datasets 1 and 2. Instead, we employed a simulation‐based approach to conduct a sensitivity power analysis to identify the minimal detectable effect size. This analysis was designed to assess the power of the given sample size to detect an interaction effect between loneliness and condition, specifically focusing on the negative episode condition, with a significance level set at *α* = .05. The findings revealed that the given sample sizes of the two datasets had medium power (Dataset 1: 1 – *β* = .608, 95% confidence interval (CI) = [.594, .622]; and Dataset 2: 1 – *β* = .602, 95% CI = [.588, .616]) to detect the interaction effect. These power estimates and CIs were derived from 5000 simulations for each dataset (see Appendix S1). The medium power level indicates a reasonable, albeit not optimal, likelihood of detecting the interaction effect, suggesting that while the data could identify significant effects, larger sample sizes could increase the power. Thus, prior to collecting Datasets 3 and 4, we conducted simulation‐based power analyses on the interaction effect using Dataset 2 with varied sample sizes (5000 simulations for each sample size). The findings indicated that a sample size of 1400 was sufficient to replicate the significant interaction effect of loneliness and the negative episode condition in Dataset 2 with statistical power 1 – *β* = .823 (95% CI = [.812, .834]) and *α* = .05 (see Appendix S1 for details). Therefore, we pre‐registered the expected sample size, analysis plan and expected results (based on the findings of Dataset 2) and planned to recruit 1600 participants for Datasets 3 and 4, considering a dropout rate of 10%.

#### Data screening

Data screening was conducted according to the following criteria. Responses completed in less than 3 minutes (4 in Dataset 2, 62 in Dataset 3 and 7 in Dataset 4) or more than 120 minutes (1 in Dataset 2, 1 in Dataset 3 and 6 in Dataset 4) were excluded from the analysis. The four datasets included one direct question scale (DQS) to detect careless responses (Maniaci & Rogge, 2014). Dataset 1 also included the instructional manipulation check (IMC; Oppenheimer et al., 2009). Participants who did not accurately respond to the DQS and IMC (3 in Dataset 1) or DQS (3 in Dataset 2, 36 in Dataset 3 and 42 in Dataset 4) were removed from the analysis. Additionally, participants who did not agree to write an essay on a given topic (5 in Dataset 1, 9 in Dataset 2, 80 in Dataset 3 and 69 in Dataset 4) or to use their responses for the analysis (15 in Dataset 1) were excluded. Answers containing meaningless content (repetition of the same characters or phrases, etc.) or missing values for psychological variables were also removed (2 in Dataset 1, 1 in Dataset 2, 40 in Dataset 3 and 18 in Dataset 4).^3^ Finally, Dataset 1 included 497 responses (303 women and 194 men; mean age = 37.9 years, *SD* = 9.87); Dataset 2 included 549 responses (252 women, 291 men and 6 other/unknown; mean age = 42.7 years, *SD* = 10.1); Dataset 3 included 1499 responses (455 women, 1016 men and 28 other/unknown; mean age = 50.2 years, *SD* = 11.7); and Dataset 4 included 1550 responses (445 women, 1086 men and 19 other/unknown; mean age = 51.0 years, *SD* = 11.9).

### Procedure

Following the existing research design when Dataset 1 was collected (Igarashi et al., 2022), the online experiments used a between‐participant design (emotional valence of an episode: positive, negative and neutral). Participants completed an experiment on Qualtrics via personal computers. After reporting demographic information, participants were randomly allocated to a condition in which they were asked to recall ‘a very happy episode from the recent past’ (positive episode condition), ‘a very tough episode from the recent past’ (negative episode condition) or ‘an episode from an ordinary day when nothing special happened in the recent past’ (neutral episode condition) and concretely explain the contents by imagining their felt emotion as vividly as possible. The words ‘recent past’ were replaced with ‘last three months’ in Dataset 4 to adjust the term to life event measures (see below). Subsequently, participants were asked to proceed with the topic or withdraw (this was because of ethical considerations for the arousal of negative emotions online). If participants agreed to continue, they were instructed to describe an episode related to the topic in at least 200 Japanese characters.^4^ Sample episodes of high‐ and low‐lonely individuals in each condition are presented in Appendix S1.

Upon completion of the episode writing, participants provided responses pertaining to the emotional valence of the episode (one item: ‘How was the experience for you?’ ranging from ‘1: very negative’ to ‘6: very positive’), the impact of the episode (two items: ‘I often remember the experience’ and ‘The experience was important for me’ ranging from ‘1: strongly disagree’ to ‘6: strongly agree’; αs ≥ .738 and ωs ≥ .738 across the datasets) and the current positive and negative mood states (measured by the Japanese version of Positive and Negative Affect Schedule [PANAS] Scale; 16 items; positive emotion: αs ≥ .905 and ωs ≥ .906; negative emotion: αs ≥ .927 and ωs ≥ .929; Sato & Yasuda, 2001) on 6‐point Likert scales (‘1: strongly disagree’ to ‘6: strongly agree’). Participants also rated loneliness, measured by the revised version of the University of California, Los Angeles Loneliness Scale (20 items; αs ≥ .943 and ωs ≥ .945) on 4‐point Likert scale (‘1: I never feel this way’ to ‘4: I often feel this way’) in Japanese (Kudoh & Nishikawa, 1983), after recalling an episode in Datasets 1 and 4 or before recalling an episode in Datasets 2 and 3.^5^ The reliabilities of the psychological scales in each dataset are presented in Appendix S1.

In Dataset 4, participants also answered depression and life events scales as control variables. Depression was measured by the 10‐item short form of the Center for Epidemiologic Studies Depression Scale (CESD‐10; 10 items; *α* = .886 and *ω* = .892; Andresen et al., 1994) on a 4‐point Likert scale (‘0: less than one day’ to ‘3: five to seven days’); and Japanese items were adapted from Shima et al. (1985). Life events in the last 3 months were measured by the Scale of Life Events in Interpersonal and Achievement Domains (15 items drawn from Takahira, 1998) on a dichotomous scale (‘0: did not experience’ or ‘1: experienced’). Five items served for positive individual events (e.g. ‘The plan I had made went as planned’; *α* = .849 and *ω* = .856); five items for positive social events (e.g. ‘I enjoyed chatting with family, partners, friends, and colleagues’; *α* = .791 and *ω* = .797); and five items for negative social events (e.g. ‘I had an argument with family, partners, friends, and colleagues’; *α* = .730 and *ω* = .741).

### Text analysis

Unlike English, the Japanese language lacks spaces between words, making the identification of word boundaries by word segmentation necessary for text analysis. The text data from the written episodes were pre‐processed and tokenized by MeCab (Kudo, 2006) and IPAdic (Asahara & Matsumoto, 2003), and analysed by the J‐LIWC2015 dictionary (Igarashi et al., 2022). MeCab is a word segmentation tool for Japanese text. IPAdic is a Japanese lexical database created for the segmentation and tokenization of Japanese text strings into individual words. The J‐LIWC2015 is the Japanese version of the LIWC2015 dictionary and is capable of quantifying linguistic and psychological characteristics of Japanese words by validated categories. We specifically focused on social words included in the social process category (e.g. ‘meet’, ‘talk’, ‘family’ and ‘friends’) in the LIWC (Pressman & Cohen, 2007) for subsequent analyses.

### Data analysis

For the Likert‐type psychological scales that include multiple items, the mean score (total score divided by the number of items) was used in the analysis. First, a series of 4 (Dataset) × 3 (episode: positive, negative and neutral) analyses of variance (ANOVAs) were conducted on the loneliness and manipulation check variables, followed by multiple comparisons (using Holm's method for *p*‐value adjustments) when the main or interaction effect was significant.

We then fit a generalized linear model to each dataset using a negative binomial distribution with a log‐link function to examine how the use of social words was associated with loneliness scores under different emotional valences. The negative binomial distribution is commonly used in natural language processing to calculate the frequency of a word's appearance in a group of documents (Bernauer et al., 2018; Katz, 1996), and helps signify the difference between words that are likely to signal what the author is thinking about (i.e. social words in this study) and words that are not; the less often a word appears in all the documents, the more likely it is to be important for understanding a specific document written by a specific author. This is because important words are usually unique to a certain topic or author and appear less frequently than other words such as articles (‘a’ or ‘the’) in English and postpositional particles (‘*no*’ or ‘*ga*’) in Japanese.

The current model, designed to predict the frequency of social words as the dependent variable, included the loneliness score (centred by means in each condition) and two dummy‐coded variables for the emotional valence of episode (the positive and negative episode conditions [coded 1 for each]; the neutral episode condition was set as a baseline [coded 0]), and two interaction variables between loneliness scores and emotional valence and gender (man, woman and other) were set as control variables. Originally unintended, the decision to include the gender variable in the model was made after exploratory analyses revealed a significant effect of gender across all four datasets. Given the variance in the length of each essay, the total number of words in each essay was log‐transformed to align with the link function, and this transformed measure was included as an offset term. In Dataset 4, we also conducted an analysis including the control variables (depression and life events).

Finally, we performed a meta‐analysis across the four datasets. One of the strengths of the current study lies in utilizing multiple large datasets, which employ the same methods to investigate the same question, a process further enriched by conducting a meta‐analysis. A meta‐analysis method processes individual‐level data (often called one‐stage individual‐patient data) from multiple datasets simultaneously by a generalized linear mixed model with random effects to accommodate the clustering of individuals within the datasets (Cumming, 2014). We added a random intercept representing datasets to the model and analysed the whole data (*N* = 4095).

## RESULTS

All analyses were conducted using R Version 4.3.2 (R Core Team, 2023).

### Manipulation check

**FIGURE 1:**
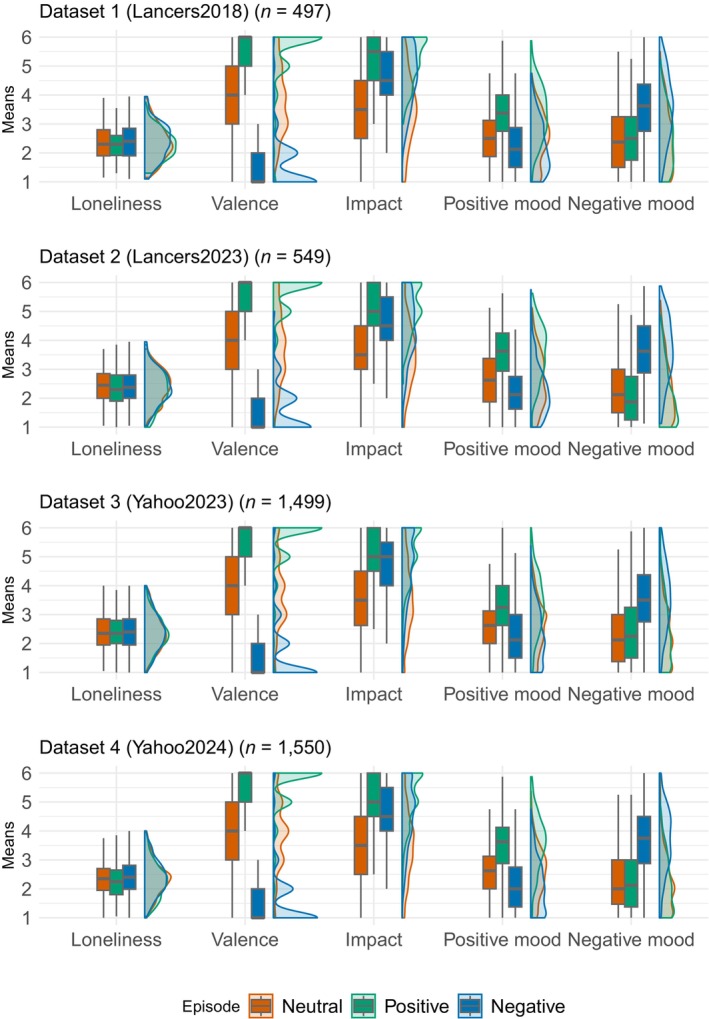
Box and density plots of loneliness, emotional valence, impact of episodes and positive and negative mood states. Loneliness ranges from 1 (never) to 4 (often). Emotional valence ranges from 1 (very negative) to 6 (very positive), in which the midpoint is 3.5 (1 + 6/2). Impact, positive and negative mood ranges from 1 (strongly disagree) to 6 (strongly agree).

Figure 1 depicts box and density plots of loneliness and manipulation check variables. All four datasets displayed consistent patterns in loneliness and the experimental manipulation of emotional valence in episodes (see Appendix S1 for details). No significant difference in loneliness was found across datasets (*p* = .118), measured after (Datasets 1 and 4) or before (Datasets 2 and 3) the manipulation. The level of loneliness in the positive episode condition was lower than in the negative and neutral episode conditions (*p*s < .01). This would be because of the procedure of participants' allocation to each condition, in which we accepted withdrawals after participants confirmed the topic of episodes to write. The emotional valence of the episode was most positive in the positive episode condition, followed by the neutral and negative episode conditions (*p*s < .001). The impact of the episode in the positive and negative episode conditions was higher than that in the neutral episode condition (*p*s < .001).

One‐sample *t*‐tests also indicated that the mean of the emotional valence was significantly higher than the midpoint of the emotional valence scale (3.5 on a 6‐point Likert scale) in the neutral and positive episode conditions and was significantly lower than the midpoint (*p*s < .002) in the negative episode condition. The level of positive mood was significantly higher in the positive episode condition than in the neutral and negative episode conditions (*p*s < .001), and the level of positive mood in Dataset 4 was lower than in Datasets 2 and 3 (*p*s < .05). Contrastingly, the level of negative mood was significantly higher in the negative episode condition than in the neutral and positive episode conditions (*p*s < .001), and the level of negative mood in the neutral condition was higher than in the positive condition in Dataset 2 and lower than in the positive condition in Dataset 3 (*p*s < .05). The findings indicate that the manipulation was successful across the datasets.

### Modelling social word use

#### Descriptive statistics

Prior to the model‐based analysis, we summarize the relationship between loneliness and the use of social words in each condition descriptively. Social words as a proportion of the total number of words in the episode did not show significant rank correlations with loneliness in the negative episode condition (Dataset 1: *ρ* = .009, 95% CI = [−.139, .155], *p* = .909; Dataset 2: *ρ* = .037, 95% CI = [−.106, .178], *p* = .612; Dataset 3: *ρ* = .011, 95% CI = [−.079, .101], *p* = .812; Dataset 4: *ρ* = −.020, 95% CI = [−.110, .070], *p* = .664). Conversely, significant negative correlations between loneliness and the use of social words were found in the positive episode condition (Dataset 1: *ρ* = −.233, 95% CI = [−.374, −.082], *p* = .003; Dataset 2: *ρ* = −.172, 95% CI = [−.308, −.023], *p* = .019; Dataset 3: *ρ* = −.218, 95% CI = [−.302, −.131], *p* < .001; Dataset 4: *ρ* = −.167, 95% CI = [−.249, −.083], *p* < .001). The neutral episode condition yielded significant negative correlations except Dataset 3 (Dataset 1: *ρ* = −.234, 95% CI = [−.377, −.081], *p* = .003; Dataset 2: *ρ* = −.220, 95% CI = [−.358, −.073], *p* = .004; Dataset 3: *ρ* = −.070, 95% CI = [−.153, .014], *p* = .103; Dataset 4: *ρ* = −.133, 95% CI = [−.021, −.050], *p* = .002).

#### Parameter estimation and simple slope analysis

**FIGURE 2:**
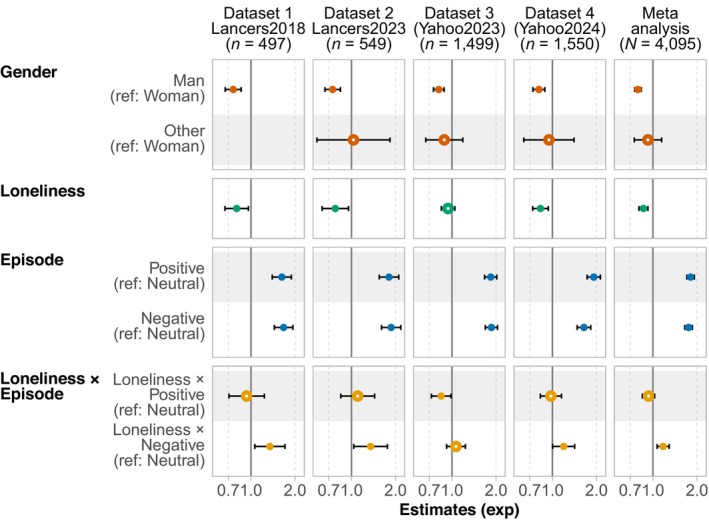
Estimated results of generalized linear models on the use of social words. The model used negative binomial distribution with log link and included the total number of words as an offset term. The estimates were exponentiated. Error bars represent 95% confidence intervals. Filled (●) and unfilled (○) circles represent significant (*p* < .05) and non‐significant estimates respectively. Baseline (reference) categories were the neutral episode condition for episode and woman for gender. In Dataset 1, the ‘other’ choice in the gender item was not available. Loneliness was mean centred in each condition (episode). Detailed information about the estimated results (e.g. coefficients, standard errors and exact *p*‐values) is reported in Appendix S1.

Figure 2 shows parameter estimates of the generalized linear models (after exponentiation) on social word use in each dataset. The effect of gender was consistent across all datasets, showing a tendency for men to use social words less frequently than women (baseline). The coefficients of the dummy‐coded variables of emotional valence were significantly greater than 1 in the exponentiated scale for both positive and negative episode conditions across the datasets, showing that individuals used more social words on average when asked about positive and negative episodes rather than neutral episodes (baseline). In Datasets 1, 2 and 4, the exponentiated coefficients of loneliness were significantly smaller than 1, indicating that an increase in loneliness was associated with a proportionate decrease in the expected count of social words. Additionally, the significant interaction effect between the negative episode condition and loneliness moderated the trend in these datasets, indicating that the relationship between loneliness and social word use varied between neutral episodes (baseline) and negative episodes. In Dataset 3, the effect of loneliness in the neutral episode condition was not significant; however, a significant interaction effect was observed between the positive episode condition and loneliness. The findings indicate that the simple slopes of loneliness differ based on the type of episodes.

Then, we estimated the slopes of loneliness in the neutral, positive and negative episode conditions. The results are presented in Table 1. Across the four datasets, the simple slopes of loneliness on social word use did not significantly differ from zero in the negative episode condition. However, the neutral and positive episode conditions yielded significant negative simple slopes in three of the four datasets.

**TABLE 1:** Estimate of loneliness slope on social word use in each condition.

	Episode	Estimate (slope)	*SE*	*z*	*p*	95% CI
Dataset 1 (Lancers2018) (*n* = 497)	Neutral	−1.227	0.526	−2.333	.020	[−2.258, −0.196]
Dataset 2 (Lancers2023) (*n* = 549)		−1.139	0.488	−2.335	.020	[−2.095, −0.183]
Dataset 3 (Yahoo2023) (*n* = 1499)		−0.223	0.199	−1.119	.263	[−0.613, 0.168]
Dataset 4 (Yahoo2024) (*n* = 1550)		−0.577	0.195	−2.956	.003	[−0.959, −0.194]
Meta‐analysis (*N* = 4095)		−0.569	0.140	−4.075	.000	[−0.842, −0.295]
Dataset 1 (Lancers2018) (*n* = 497)	Positive	−2.426	0.906	−2.678	.007	[−4.201, −0.65]
Dataset 2 (Lancers2023) (*n* = 549)		−1.101	0.606	−1.816	.069	[−2.29, 0.087]
Dataset 3 (Yahoo2023) (*n* = 1499)		−1.490	0.377	−3.956	.000	[−2.229, −0.752]
Dataset 4 (Yahoo2024) (*n* = 1550)		−1.267	0.353	−3.587	.000	[−1.96, −0.575]
Meta‐analysis (*N* = 4095)		−1.486	0.250	−5.937	.000	[−1.976, −0.995]
Dataset 1 (Lancers2018) (*n* = 497)	Negative	0.705	0.762	0.926	.354	[−0.788, 2.199]
Dataset 2 (Lancers2023) (*n* = 549)		0.377	0.680	0.554	.579	[−0.956, 1.711]
Dataset 3 (Yahoo2023) (*n* = 1499)		0.017	0.362	0.047	.962	[−0.692, 0.726]
Dataset 4 (Yahoo2024) (*n* = 1550)		−0.060	0.326	−0.184	.854	[−0.698, 0.579]
Meta‐analysis (*N* = 4095)		0.075	0.230	0.329	.742	[−0.375, 0.526]

Abbreviations: CI, confidence interval; *SE*, standard error.

#### Estimation including the control variables

Finally, we analysed Dataset 4 by adding the control variables of depression and life events to the model (see Appendix S1 for details). The full model included many terms regarding psychological and life event variables (i.e. 5 [loneliness, depression, positive individual life events, positive social life events and negative social life events]) and their interactions with emotional valence (5 × 2 [positive/negative episode conditions] = 10 interaction effects) to explain social word use; therefore, we performed a stepwise model selection by Akaike's information criterion (AIC). The final model included gender, positive individual life events, positive social life events and negative social life events as significant control variables, in addition to loneliness, emotional valence and their interactions. Consistent with the above findings without the control variables, the final model revealed that the slope of the loneliness score in the negative episode condition was not significant (slope = 0.255, standard error (*SE*) = 0.361, 95% CI = [−0.453, 0.963], *z* = 0.705, *p* = .481), whereas those in the neutral and positive episode conditions were significant and negative (slope = −0.416, *SE* = 0.212, 95% CI = [−0.830, −0.001], *z* = −1.964, *p* = .0495, in the neutral episode condition and slope = −1.010, *SE* = 0.383, 95 % CI = [−1.761, −0.258], *z* = −2.634, *p* = .008, in the positive episode condition). These results indicate that loneliness influenced the use of social words in episode writing, even after controlling for event exposure.

### Meta‐analysis

**FIGURE 3:**
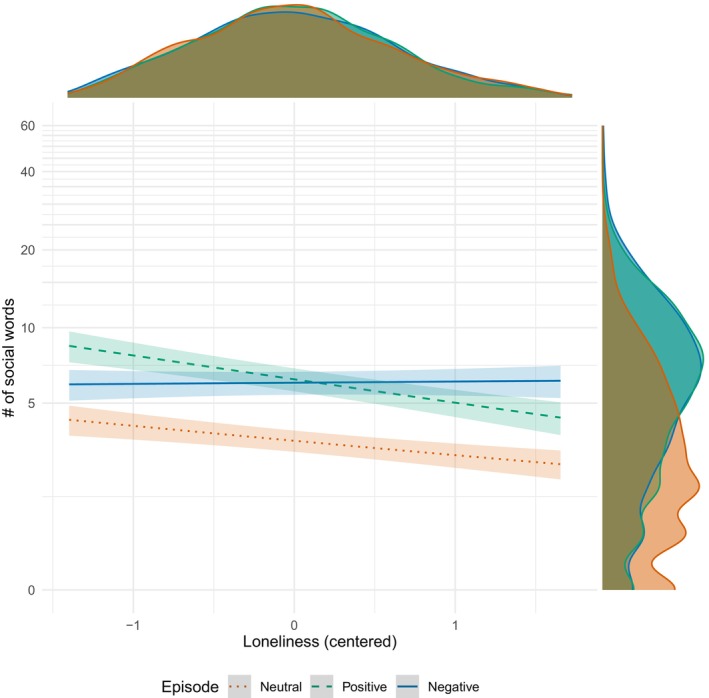
Association between loneliness and the use of social words in meta‐analysis of four datasets (*N* = 4095). Margins indicate 95% confidence intervals for the predicted values of each regressed line. Density plots are presented on top (loneliness) and left (# of social words). The generalized linear mixed model (negative binomial distributions with log link) included the total number of words in each episode as an offset term and dataset as a random intercept. Loneliness was mean centred in each condition (episode).

In the meta‐analysis model, τ represents an index of dataset heterogeneity, defined as the standard deviation of a random intercept. The estimated τ was .097, 95% CI = [0.051, 0.244], indicating that the use of social words did not vary substantially across the datasets. As shown in Figure 2, the meta‐analysis replicated the same patterns with the analyses in each dataset (see Appendix S1 for estimated values). The exponentiated coefficient of loneliness in the neutral episode condition was significant and smaller than 1, whereas the interaction coefficient of loneliness and the negative episode condition was significant and greater than 1. Table 1 indicates that the slope of the loneliness score on social word use was not significant in the negative episode condition but significant and negative in the positive episode condition. The predicted values are presented in Figure 3 based on the parameter estimates in the neutral, positive and negative episode conditions. Overall, the results indicate that the meta‐analysis confirmed the findings to be consistent across studies.

The overall patterns partially indicate negative memory biases for loneliness. Namely, high‐lonely individuals were less likely to use social words in their positive episodes. However, individuals tended to use social words in their negative episodes regardless of their level of loneliness. The findings did not indicate the presence of general social memory biases among high‐lonely individuals.

## DISCUSSION

The current study examined how chronic loneliness is related to the use of social words in autobiographical memories with emotional valence. The findings reveal that high‐lonely individuals exhibit a decline in the use of social words in positive episodes compared with low‐lonely individuals, partially suggesting a negative memory bias. This pattern was also evident in the neutral episode condition. Unlike the previous studies by Gardner et al. (2000, 2005), we did not find evidence of general social memory biases among high‐lonely individuals irrespective of the emotional valence of episodes. In addition, the level of loneliness was not significantly related to the use of social words in the negative episode condition. These patterns were observed even when controlling for the experience of positive and negative social events.

The absence of positive memories is distinguishable from the presence of negative memories (Crowe & Higgins, 1997). High‐lonely individuals exhibit less activation in the brain reward systems during positive situations (Cacioppo et al., 2009) and do not engage in thoughtful responses to positive interpersonal stimuli, such as touching (Saporta et al., 2021). An experience sampling study also revealed that higher loneliness leads to lower momentary positive emotions in daily life (Kuczynski et al., 2022; van Roekel et al., 2014). Autobiographical memory may reflect daily experiences where high‐lonely individuals unconsciously avoid positive social cues and engage with others in a way that perpetuates their sense of isolation.

Notably, there was no significant difference in the use of social words in the negative episode condition by the level of loneliness. This finding is novel and predicted by neither negative nor social memory biases nor trait‐mood congruency effects (Rusting, 1998). One explanation is that recalling a certain degree of negative interpersonal experiences is fairly common in daily life, regardless of the level of loneliness. Interpersonal conflicts and stress are natural experiences for several individuals, frequently observed across different cultures (Hashimoto et al., 2012). Interpersonal tension is the most frequent daily stressor, followed by network stressors affecting close relationships (Almeida et al., 2002). Even close relationships such as those between families and couples are not immune to interpersonal conflicts that give rise to negative emotions (Bolger et al., 1989; Van Orden et al., 2010), and it is common for children to have distressing interpersonal experiences (Copeland et al., 2007). Consequently, the degree of recalling negative interpersonal experiences may not substantially vary as a function of loneliness.

As highlighted by Williamson et al. (2019) and Williams et al. (2022), negative autobiographical memory plays a crucial role in shaping our current actions. This memory works as a compass, providing direction and motivation for our actions. The ability to learn from past negative experiences, often viewed as an adaptive trait, enhances our capacity to effectively navigate future scenarios. In ancestral environments, where individuals reside in a small community, temporal social conflicts and disagreements among individuals can signal immediate danger, requiring alertness to these threatening events. This may explain why both chronically high‐ and low‐lonely individuals tend to look back on negative social episodes to a similar degree. An evolutionary predisposition to remember negative social interactions would be advantageous for an individual's survival irrespective of their loneliness levels.

However, cognitive resource allocation manifests differently for positive social cues. During a chronic state of loneliness, which may equate to vulnerability in evolutionary terms, allocating cognitive resources to seek positive social memories can be a ‘luxury’ that compromises immediate survival needs. This reasoning is consistent with the observed neurological data, which showed less reward system activation for high‐lonely individuals in positive situations (Cacioppo et al., 2009). The lowered neurological responses may result from evolved mechanisms that down‐regulate the importance of positive social engagement when an individual is isolated, conserving cognitive and emotional resources for immediate survival concerns.

This scarcity of positive social memories can significantly exacerbate socioemotional distress among high‐lonely individuals. Although loneliness signals individuals to re‐engage with others to alleviate their sense of isolation (see Kornienko et al., 2020), this persistent inability to recall positive interactions can lead to a deleterious cycle. High‐lonely individuals feel motivated to seek social connections; however, when remembering that past social interactions have been hard to achieve, the motivation to seek out and engage in new social relationships may diminish, leading to further withdrawal and isolation. That is, the dearth of positive social memories hinders their efforts of social reaffiliation. This cycle perpetuates chronic loneliness, making it increasingly difficult for individuals to break free from their state of loneliness.

Contrary to the initial assumption, there was no indication of general social memory biases among high‐lonely individuals. The sample sizes of previous research (Gardner et al., 2000, 2005) are smaller (*N*s < 100 in each experiment) than those in the current study; therefore, existing evidence on social memory biases regardless of emotional valence might not be as robust as initially expected. The difference in the target of recall between these studies (another person's diary) and the current study (one's experience) may have impacted the findings. However, several studies also report inconsistent evidence for hypersensitivity towards socioemotional stimuli triggered by loneliness, such as no clear association between loneliness and performance in tasks involving the facial and emotional recognition of others (Kanai et al., 2012; Lodder et al., 2016). Additionally, being excessively sensitive to all social cues may be too costly from an evolutionary perspective. An overly sensitive social monitoring system may lead to frequent false alarms, wasting the energy and cognitive resources needed for further survival. The current findings obtained from large samples with pre‐registered plans raise questions about the credibility of social memory biases irrespective of emotional valence to explain how chronically high‐lonely individuals recall social information from their personal experiences.

### Practical implications

The findings of the present study have several practical implications. Interventions targeting loneliness should not merely focus on decreasing sensitivity to negative social episodes. Rather, the absence of positive social memories appears to be a more salient issue for high‐lonely individuals. Intervention programmes may benefit from incorporating elements that guide high‐lonely individuals to recognize, seek out and appreciate positive social interactions. Specifically, practitioners could develop strategies that help individuals recall positive social experiences more effectively. For instance, incorporating memory‐enhancing techniques such as journaling positive interactions and using cognitive‐behavioural strategies to reframe social experiences positively would be beneficial.

Interventions could also be designed to include activities that reinforce the recall of positive social interactions such as group discussions in which participants share positive social experiences and guided imagery exercises that help individuals visualize positive social scenarios. By integrating these elements, intervention programmes could provide a more comprehensive approach that not only aims to increase positive interactions but also enhances the ability of high‐lonely individuals to remember and value these interactions, leading to sustained improvements in their social well‐being.

### Limitations

This study has some limitations. While we used autobiographical memory as personal and meaningful components for recall, laboratory experiments on emotion and memory have employed standardized positive and negative words as recall cues (García‐Bajos & Migueles, 2017; Rusting, 1998; Williams et al., 2007). Furthermore, for low‐lonely individuals, negative episodes involving interpersonal experiences may include something unpleasant happening to their family and friends rather than themselves, such as a disease or fatality, whereas lonely people may recall their own negative episodes in social situations. These are not distinguishable through the analysis of social word use. Future research should expand upon these limitations by utilizing a variety of stimuli for recall, ranging from standardized emotional words to more complex social scenarios. This approach allows researchers to determine whether the observed relationship between loneliness and recall of positive social experiences remains consistent across various memory cues.

Another limitation of this study is the measure of life events, which only captures the occurrence or absence of the listed events. A more comprehensive approach such as experience sampling would include an assessment of the frequency and quality of social interactions in daily lives, distinguishing between positive and negative experiences. This additional detail could better explain how different types of social experiences influence autobiographical memory in high‐ and low‐lonely individuals.

The cultural background of the Japanese sample used in this study may be considered a potential influencing factor for the findings. This study does not involve cultural psychology, and much of the existing research focusing solely on Westerners has not explicitly discussed cultural aspects of the samples (Cheon et al., 2020; Henrich et al., 2010). However, there is a possibility that cultural factors specific to Japanese samples, such as the relational nature of emotions, can influence the association between loneliness and autobiographical memory, as loneliness might serve as a signal inviting acceptance from others (Mesquita, 2022). Additionally, Japanese samples tend to exhibit prevention‐focused self‐regulation (Ouschan et al., 2007), which could diminish the differences in autobiographical memory between high‐ and low‐lonely individuals. Future research should consider collecting data from diverse populations to assess the generalizability of the findings.

## CONCLUSION

This study provides critical insights into the relationship between chronic loneliness and autobiographical memory. Based on the analysis involving large datasets, we observed no significant difference in the use of social words in negative episodes between high‐ and low‐lonely individuals. Negative social memories may serve as a basis for learning and future social navigation for all individuals, rather than specifically affecting high‐lonely individuals. The findings highlight the sparseness of social word use in positive episodes as a defining characteristic of loneliness in the context of autobiographical memory. Additionally, the findings indicate no pattern of heightened general social memory biases among chronically high‐lonely individuals as argued in previous research. These revelations have important implications for future research and psychological interventions aimed at alleviating loneliness.

## AUTHOR CONTRIBUTIONS

**Tasuku Igarashi:** Conceptualization; investigation; funding acquisition; methodology; validation; visualization; writing – original draft; project administration.

## CONFLICTS OF INTEREST

The author has no conflicts of interest to report.

## Supporting information


Appendix S1

## Data Availability

The data that support the findings of this study are openly available in the Open Science Framework at https://osf.io/s7qrg/.

## Footnotes

^1^
Igarashi (2019) developed the Japanese version of the three‐item loneliness scale by using loneliness scores and demographic and personality variables. Igarashi et al. (2022) developed the Japanese version of the Linguistic Inquiry and Word Count 2015, including a validity check experiment that compared the contents of episodes written in different moods. None of the studies handled the association of loneliness with the contents of episodes.

^2^
All research was approved by the Ethical Review Board of the author's institution (#181162).

^3^
Five participants in Dataset 3 and 25 participants in Dataset 4 primarily described their episodes in hiragana (the Japanese cursive syllabary), but the J‐LIWC2015 cannot handle that form of non‐standard Japanese writing. We used ChatGPT (GPT‐4) to convert the descriptions into standard Japanese writing composed of hiragana, katakana (the angular form of Japanese syllabary) and kanji (Chinese characters). Subsequently, all conversions were checked by two Japanese native speakers and used in the analysis.

^4^
When we asked participants to describe an episode online in Datasets 2, 3 and 4, we implemented a custom JavaScript that allowed them to copy and paste texts only within the text form. This was to prevent them from using generative artificial intelligence technologies such as ChatGPT outside Qualtrics to create an episode.

^5^
The Japanese version of the Three‐Item Loneliness Scale (Igarashi, 2019) was also applied in all datasets but not used in the analysis.
