# Supplementary material for: Loneliness and socioemotional memory
Source: Br J Soc Psychol. 2024 Jun 29;64(1):e12783. doi: 10.1111/bjso.12783 (PMC11588037; doi:10.1111/bjso.12783)
Supplement: Supplementary file 1 — Appendix S1 [file BJSO-64-0-s001.docx]

**Supplementary materials**

**Loneliness and socioemotional memory**

Table of contents

[Sensitivity power analysis for Datasets 1 and 2 2](#_Toc169694614)

[Sample size determination for Datasets 3 and 4 3](#_Toc169694615)

[Descriptive statistics 4](#_Toc169694616)

[Scale reliability 6](#_Toc169694617)

[Manipulation check 7](#_Toc169694618)

[Sample episodes in Dataset 3 10](#_Toc169694619)

[Estimates of the generalized linear models in Datasets 1 to 4 12](#_Toc169694620)

[Estimates of the generalized linear model in the meta-analysis 14](#_Toc169694621)

[Estimates of the generalized linear model including the control variables in Dataset 4 15](#_Toc169694622)

[Reference 20](#_Toc169694623)

# Sensitivity power analysis for Datasets 1 and 2

The goal of sensitivity power analysis is to understand how changes in parameters (e.g., sample size or effect size) could influence the power to detect an effect. The code posted on the Open Science Framework (<https://osf.io/s7qrg/>) simulates data based on estimated parameters from actual data (*N* = 497 in Dataset 1 and *N* = 549 in Dataset 2) and then assesses the proportion of simulations in which the interaction term of loneliness and the negative episode condition (see Table S4) is statistically significant (α = .05). Although the initial model estimation is based on actual data, the subsequent simulations to calculate power are prospective in nature and help understand the likelihood of detecting significant effects under varying sample sizes for Datasets 3 and 4, which align with the goals of sensitivity power analysis. The calculation of power and confidence intervals for these power estimates uses a forward-looking approach, assessing the robustness of future studies designed similarly to the current model.

# Sample size determination for Datasets 3 and 4

Table S1 presents the results of power analysis by a simulation (5,000 iterations) varying the sample size based on the findings of Dataset 2 containing the significant negative interaction effect between loneliness and the negative episode condition. The results indicated that a sample size of 1,400 was sufficient to replicate the findings from Dataset 2 with statistical power 1 – β = .823 (95% CI = [.812, .834]) and α = .05. Therefore, we preregistered the expected sample size and findings of Dataset 2 as a prediction for the findings of Datasets 3 and 4 and planned to recruit 1,600 participants, considering a dropout rate of 10% and the lowest 95% CI over .80.

Table S1

Results of the power analysis for Datasets 3 and 4 (α = .05)

| *N* | Power | 95% CI |
| --- | --- | --- |
| 500 | .566 | [.552, .579] |
| 600 | .629 | [.616, .642] |
| 700 | .674 | [.661, .687] |
| 800 | .703 | [.690, .716] |
| 900 | .726 | [.714, .739] |
| 1,000 | .756 | [.744, .768] |
| 1,100 | .776 | [.764, .788] |
| 1,200 | .793 | [.781, .804] |
| 1,300 | .805 | [.794, .816] |
| 1,400 | .823 | [.812, .834] |
| 1,500 | .829 | [.819, .840] |
| 1,600 | .834 | [.823, .844] |
| 1,700 | .844 | [.834, .854] |
| 1,800 | .856 | [.846, .866] |
| 1,900 | .860 | [.851, .870] |
| 2,000 | .866 | [.857, .875] |

*Note*. CI = confidence interval. Codes are available at the Open Science Framework (<https://osf.io/s7qrg/>).

# Descriptive statistics

Table S2

Means and standard deviations (in parentheses) of the variables in each dataset

|  | Episode | # of social words | Word count | Loneliness | Positive emotion | Negative emotion | Emotional valence | Impact | Depression | Positive social life event | Negative social life event | Positive individual life event |
| --- | --- | --- | --- | --- | --- | --- | --- | --- | --- | --- | --- | --- |
| Dataset 1  (Lancers2018) | Neutral | 5.554 (5.308) | 159.376 (55.843) | 2.341 (0.611) | 2.521 (0.913) | 2.487 (1.115) | 3.828 (1.241) | 3.516 (1.236) |  |  |  |  |
|  | Positive | 8.222 (5.459) | 151.568 (54.687) | 2.287 (0.506) | 3.350 (0.951) | 2.525 (1.042) | 5.377 (1.142) | 5.160 (0.791) |  |  |  |  |
|  | Negative | 9.854 (8.681) | 173.011 (84.417) | 2.402 (0.613) | 2.218 (0.890) | 3.536 (1.174) | 1.618 (0.945) | 4.593 (1.046) |  |  |  |  |
| Dataset 2  (Lancers2023) | Neutral | 4.494 (4.141) | 155.186 (56.374) | 2.415 (0.568) | 2.645 (0.993) | 2.362 (1.076) | 3.843 (1.281) | 3.532 (1.185) |  |  |  |  |
|  | Positive | 7.139 (5.820) | 139.086 (36.511) | 2.315 (0.635) | 3.561 (0.978) | 2.108 (0.999) | 5.561 (0.803) | 5.037 (0.842) |  |  |  |  |
|  | Negative | 8.095 (6.640) | 152.921 (67.326) | 2.393 (0.589) | 2.263 (0.898) | 3.624 (1.088) | 1.679 (0.883) | 4.408 (1.134) |  |  |  |  |
| Dataset 3  (Yahoo2023) | Neutral | 3.723 (3.960) | 135.575 (40.403) | 2.418 (0.637) | 2.609 (0.927) | 2.335 (1.059) | 3.817 (1.324) | 3.560 (1.276) |  |  |  |  |
|  | Positive | 6.418 (4.660) | 130.471 (28.188) | 2.367 (0.594) | 3.322 (1.019) | 2.473 (1.176) | 5.450 (1.008) | 5.028 (0.929) |  |  |  |  |
|  | Negative | 7.040 (5.233) | 139.745 (44.315) | 2.407 (0.654) | 2.265 (0.921) | 3.569 (1.157) | 1.726 (1.142) | 4.577 (1.202) |  |  |  |  |
| Dataset 4  (Yahoo2024) | Neutral | 3.107 (3.152) | 134.455 (34.031) | 2.361 (0.621) | 2.579 (0.889) | 2.265 (1.052) | 3.909 (1.258) | 3.526 (1.236) | 0.900 (0.643) | 2.960 (1.569) | 1.480 (1.405) | 3.286 (1.115) |
|  | Positive | 6.051 (5.392) | 137.559 (61.904) | 2.273 (0.627) | 3.538 (0.992) | 2.274 (1.021) | 5.443 (0.956) | 5.071 (0.924) | 0.778 (0.591) | 3.186 (1.518) | 1.460 (1.437) | 3.348 (1.104) |
|  | Negative | 5.290 (5.093) | 140.148 (42.576) | 2.409 (0.619) | 2.129 (0.882) | 3.648 (1.114) | 1.661 (1.084) | 4.532 (1.124) | 1.010 (0.670) | 3.044 (1.560) | 1.659 (1.434) | 3.169 (1.172) |

# Scale reliability

Table S3

Reliability of the scales

|  | Dataset 2  (Lancers2023) | |  | Dataset 3  (Yahoo2023) | |  | Dataset 4  (Yahoo2024) | |
| --- | --- | --- | --- | --- | --- | --- | --- | --- |
|  | α | ω |  | α | ω |  | α | ω |
| Loneliness | .943 | .945 |  | .946 | .947 |  | .947 | .949 |
| Impact | .738 | .738 |  | .792 | .792 |  | .771 | .771 |
| Positive emotion | .909 | .912 |  | .905 | .906 |  | .910 | .911 |
| Negative emotion | .932 | .933 |  | .932 | .933 |  | .927 | .929 |
| Depression |  |  |  |  |  |  | .886 | .892 |
| Positive social life event |  |  |  |  |  |  | .849 | .856 |
| Negative social life event |  |  |  |  |  |  | .791 | .797 |
| Positive individual life event |  |  |  |  |  |  | .730 | .741 |

*Note.* α = Cronbach’s alpha coefficients. ω = McDonald’s omega coefficients. As Dataset 1 is obtained as aggregated variables, the reliability coefficients are not calculated.

# Manipulation check

Outputs are available at the Open Science Framework (<https://osf.io/s7qrg/>).

***Analysis of variance***

Regarding loneliness and manipulation check variables, 4 (Dataset) × 3 (Episode: positive, negative, and neutral) analyses of variance were performed. Post-hoc tests indicated adjusted *p*-values based on Holm’s sequentially rejective Bonferroni procedure. Analyses were conducted by the *anovakun* function (Version 4.8.9) in R (Iseki, 2023).

**Loneliness.** The main effect for Dataset was not significant, *F* (3, 4083) = 1.961, *p* = .118, η²_p_ = .001, as was the case with the interaction effect, *F* (6, 4083) = 0.713, *p* = .713, η²_p_ < .001. There was a significant main effect for Episode with a very small effect size, *F* (2, 4083) = 6.469, *p* = .002, η²_p_ = .003. Post-hoc tests indicated that the mean score of loneliness for the positive episode condition (*M* = 2.311) was significantly lower than in the negative (*M* = 2.403, *p* = .002) and neutral (*M* = 2.384, *p* = .014) episode conditions. There was no significant difference in loneliness between the neutral and negative episode conditions (*p* = .490).

**Emotional valence.** A significant main effect for Episode was found, *F* (2, 4083) = 3013.699, *p* < .001, η²_p_ = .596. Post-hoc tests indicated that the mean score of emotional valence (higher scores indicate positive emotional valence) was significantly higher in the positive episode condition (*M* = 5.458) than in the negative episode condition (*M* = 1.671, *p* < .001), and the mean score in the neutral episode condition (*M* = 3.849) was significantly higher than in the negative episode condition (*p* < .001) and lower than in the positive episode condition (*p* < .001). Additionally, the mean score in the positive episode condition was significantly higher than in the neutral episode condition (*p* < .001). Neither the main effect of Dataset, *F* (3, 4083) = 0.577, *p* = .630, η²_p_ < .001 nor the interaction effect, *F* (6, 4083) = 0.712, *p* = .640, η²_p_ = .001, was significant.

**Impact.** A significant main effect for Episode was observed, *F* (2, 4083) = 506.512, *p* < .001, η²_p_ = .199. Post-hoc tests indicated that the mean score of impact in the positive episode condition (*M* = 5.074) was significantly higher than in the neutral (*M* = 3.533, *p* < .001) and negative (*M* = 4.527, *p* < .001) episode condition. The mean score in the negative episode condition was significantly higher than in the neutral episode condition (*p* < .001). There were no significant findings found for Dataset, *F* (3, 4083) = 0.719, *p* = .541, η²_p_ = .001, and the interaction effect, *F* (6, 4083) = 0.590, *p* = .738, η²_p_ = .001.

**Positive emotion.** A significant main effect for Episode was found, *F* (2, 4083) = 465.115, *p* < .001, η²_p_ = .186. Post-hoc tests for Episode demonstrated that the mean score of positive emotion in the positive episode condition (*M* = 3.443) was significantly higher than in the negative (*M* = 2.219, *p* < .001) and neutral (*M* = 2.589, *p* < .001) episode conditions. The mean score of positive emotion in the negative episode condition was significantly lower than in the neutral episode condition (*p* < .001). The main effect for Dataset was not significant, *F* (6, 4083) = 1.798, *p* = .145, η^2^_p_ = .001, but there was a significant interaction effect between Dataset and Episode, *F* (6, 4083) = 3.358, *p* = .003, η²_p_ = .005, although the effect size was very small. The simple effects analysis revealed significant differences in the mean scores of positive emotion across episodes across the datasets. In Dataset 3 (Yahoo2023), the positive episode condition exhibited significantly lower mean scores of positive emotion compared with Datasets 2 (Lancers2023) and 4 (Yahoo2024) (*p* < .001).

**Negative emotion.** Significant effects were found for Episode, *F* (2, 4083) = 450.543, *p* < .001, η^2^_p_ = .181. Whereas the main effect of Dataset was not significant, *F* (6, 4083) = 2.532, *p* = .055, η^2^_p_ = .002, the interaction effect between Dataset and Episode was significant with a small effect size, *F* (6, 4083) = 3.625, *p* = .001, η^2^_p_ = .005. The post-hoc analyses revealed that significantly higher mean scores of negative emotion were observed in the negative (*M* = 3.594) episode condition than in the positive (*M* = 2.345, *p* < .001) and neutral (*M* = 2.362, *p* < .001) episode conditions. Although the effect size was very small, the multiple comparisons based on the interaction effect showed that the mean score of negative emotion in the positive episode condition was higher than in the neutral episode condition (*p* < .001) in Dataset 2 (Lancers2023), but lower than in Dataset 3 (Yahoo2023). In Datasets 1 (Lancers2018) and 4 (Yahoo2024), no significant difference was found in the mean score of negative emotion between the neutral and positive episode conditions.

***One-sample t-tests***

One-sample *t*-tests were conducted to examine emotional valence across neutral, negative, and positive episodes. Higher values indicate positive emotional valence. The midpoint of the emotional valence scale (3.5 on a 6-point Likert scale) was used as the reference point for comparison. All *p*-values were adjusted by Holm’s procedure. In the neutral episode condition, participants reported a mean emotional valence ranging from 3.82 to 3.91 across datasets, which were all significantly higher than the midpoint, *t*s (156–551) = 3.31–7.65, *p* < .002. Effect sizes, Cohen’s *d*, ranged from 0.239 to 0.326, indicating small to moderate effects. In the positive episode condition, participants reported significantly higher emotional valence scores ranging from 5.38 to 5.56 across datasets, compared with the midpoint, *t*s (161–526) = 20.9–46.6, *p*s < .001, with large effect sizes ranging from 1.64 to 2.03. In the negative episode condition, participants reported significantly lower emotional valence scores ranging from 1.62 to 1.73 across datasets, compared with the midpoint, *t*s (177–474) = -26.6 to -36.8, *p*s < .001, with large negative effect sizes ranging from -1.55 to -2.06.

# Sample episodes in Dataset 3

1. High-lonely individuals
   1. Neutral episode

*I woke up in the morning, went to the bathroom, changed my clothes, ate a meal, did some searches on the internet, and watched some videos without going outside because it was a holiday with no particular plans, and then I realized it was noon. I was soaked in melancholy while randomly watching TV, played games to distract myself, and found myself eating snacks while watching a baseball game at night. The day ended when the team I was rooting for lost and I felt empty. That was the end of the day.*

- 1. Negative episode

*A friend I’ve been close to for over 20 years, has lost touch with me suddenly. No matter how many emails I sent, I never heard back from him for months. I’m so shocked and depressed. I think about the reason, but I can’t think of anything in particular (I tried to think carefully before sending messages), so I was wondering if it might be because he had been exposed to the coronavirus several times, tested positive again, and is currently sick. I have been bothered by the thought.*

- 1. Positive episode

*I read a news article on the internet about a popular 100-yen outdoor gadget and liked it so much that I walked all the way to the nearest 100-yen store. I was able to purchase the product I was looking for at the store and was also able to purchase a related product that was newly on sale at the same time. I also went to another 100-yen store nearby, and although it was not for 100 yen, they sold a product I had wanted for a long time that I could use in my vegetable garden. After much deliberation, I bought and used it.*

1. Low-lonely individuals
   1. Neutral episode

*I wake up in the morning with my children, eat breakfast, go outside with them to water the vegetables, make things out of dirt for fun, go shopping with my family, and at noon, have lunch or dessert at a family or fast-food restaurant or food court. After lunch, I take a nap with the children and spend the rest of the day watching TV and relaxing. I eat dinner with my children, then play with them again, take a bath, and go to bed. That’s a day.*

- 1. Negative episode

*I had a date with my girlfriend, but I was running late because of work, so I called her to let her know I would be late. It would have been fine if I had just made her wait, but being late meant that I missed out on a certain experience that I had planned, and she pointed out my lack of foresight in that regard. It was definitely my fault for being late, but I could not miss work, and I felt bad for her as well, so I felt like I was on the wrong side of both parties, which was exhausting and very hard.*

- 1. Positive episode

*There are always friends nearby who are willing to talk to me when I have a business problem or feel vulnerable in my personal life. I’m blessed with people who are willing to advise me and suggest solutions. I’m also very grateful for the good relationship I have with my clients. I’m extremely grateful for people always nearby who can take me out when I feel stressed. Such relationships are definitely good for my mental health. Another happy event was that a business owner who was forced into bankruptcy owing to the coronavirus pandemic has recently made an energetic comeback.*

*Note.* High- and low-lonely individuals were determined by the 20% quantiles of the loneliness score in Dataset 3. All texts were originally written in Japanese and translated into English.

# Estimates of the generalized linear models in Datasets 1 to 4

Table S4

Model estimates (Datasets 1 to 4)

|  | **Dataset 1** | | | |  | **Dataset 2** | | | |  | **Dataset 3** | | | |  | **Dataset 4** | | | |
| --- | --- | --- | --- | --- | --- | --- | --- | --- | --- | --- | --- | --- | --- | --- | --- | --- | --- | --- | --- |
|  | **(Lancers2018)** | | | |  | **(Lancers2023)** | | | |  | **(Yahoo2023)** | | | |  | **(Yahoo2024)** | | | |
|  | **(*n* = 497)** | | | |  | **(*n* = 549)** | | | |  | **(*n* = 1,499)** | | | |  | **(*n* = 1,550)** | | | |
|  | Exp (b) | *SE* | 95% CI | *p* |  | Exp (b) | *SE* | 95% CI | *p* |  | Exp (b) | *SE* | 95% CI | *p* |  | Exp (b) | *SE* | 95% CI | *p* |
| (Intercept) | 0.037 | 0.002 | [0.033, 0.042] | **< .001** |  | 0.002 | 0.002 | [0.029, 0.037] | **<.001** |  | 0.031 | 0.001 | [0.028, 0.033] | **< .001** |  | 0.026 | 0.001 | [0.024, 0.029] | **< .001** |
| Gender (man) | 0.756 | 0.048 | [0.667, 0.857] | **< .001** |  | 0.046 | 0.046 | [0.659, 0.841] | **<.001** |  | 0.813 | 0.035 | [0.747, 0.884] | **< .001** |  | 0.808 | 0.039 | [0.736, 0.887] | **< .001** |
| Gender (other) |  |  |  |  |  | 0.303 | 0.082 | [0.580, 1.834] | .912 |  | 0.886 | 0.132 | [0.662, 1.186] | .417 |  | 0.947 | 0.191 | [0.637, 1.406] | .785 |
| Loneliness | 0.798 | 0.075 | [0.664, 0.960] | **.017** |  | 0.082 | 0.141 | [0.630, 0.955] | **.019** |  | 0.941 | 0.051 | [0.846, 1.047] | .262 |  | 0.829 | 0.052 | [0.733, 0.937] | **.003** |
| Positive episode | 1.624 | 0.125 | [1.397, 1.889] | **< .001** |  | 0.141 | 0.145 | [1.548, 2.105] | **<.001** |  | 1.840 | 0.090 | [1.671, 2.025] | **< .001** |  | 1.916 | 0.102 | [1.726, 2.126] | **< .001** |
| Negative episode | 1.671 | 0.125 | [1.443, 1.934] | **< .001** |  | 0.145 | 0.150 | [1.603, 2.174] | **<.001** |  | 1.860 | 0.091 | [1.690, 2.047] | **< .001** |  | 1.642 | 0.090 | [1.475, 1.828] | **< .001** |
| Loneliness × Positive episode | 0.934 | 0.133 | [0.707, 1.234] | .633 |  | 0.150 | 0.183 | [0.846, 1.440] | .525 |  | 0.844 | 0.066 | [0.724, 0.985] | **.032** |  | 0.976 | 0.083 | [0.827, 1.154] | .779 |
| Loneliness × Negative episode | 1.346 | 0.163 | [1.062, 1.707] | **.014** |  | 0.183 | 0.303 | [1.037, 1.761] | **.033** |  | 1.066 | 0.080 | [0.919, 1.235] | .399 |  | 1.193 | 0.106 | [1.003, 1.419] | **.047** |

*Note*. *SE* = standard error, CI = confidence interval. The model used negative binomial distribution with log link and included the total number of words as an offset term. The estimates were exponentiated. Baseline categories were the neutral episode condition for episode and woman for gender. In Dataset 1, the “other” choice in the gender item was not available. Loneliness was mean centered in each condition (group).

# Estimates of the generalized linear model in the meta-analysis

Table S5

Model estimates in the meta-analysis

|  | Meta-analysis (*N* = 4,095) | | | |
| --- | --- | --- | --- | --- |
|  | Exp (b) | *SE* | 95% CI | *p* |
| Fixed effects |  |  |  |  |
| (Intercept) | 0.031 | 0.002 | [0.028, 0.035] | **< .001** |
| Sex (man) | 0.790 | 0.021 | [0.751, 0.832] | **< .001** |
| Sex (other) | 0.925 | 0.101 | [0.746, 1.146] | .475 |
| Loneliness | 0.861 | 0.031 | [0.803, 0.923] | **< .001** |
| Positive episode | 1.808 | 0.055 | [1.703, 1.919] | **< .001** |
| Negative episode | 1.752 | 0.053 | [1.650, 1.859] | **< .001** |
| Loneliness × Positive episode | 0.933 | 0.046 | [0.847, 1.028] | .161 |
| Loneliness × Negative episode | 1.174 | 0.057 | [1.067, 1.291] | **.001** |
| Random effects |  |  |  |  |
| σ^2^ | 3.24 |  |  |  |
| τ_00_ dataset | 0.01 |  |  |  |
| *N* dataset | 4 |  |  |  |

*Note*. *SE* = standard error, CI = confidence interval. The model used a negative binomial distribution with a log link and included the total number of words as an offset term and dataset as a random intercept. The estimates were exponentiated. Baseline categories were the neutral episode condition for episode and woman for gender. Loneliness was mean centered in each condition (group).

# Estimates of the generalized linear model including the control variables in Dataset 4

Table S6

Results of the stepwise model selection in Dataset 4

|  | Model 1 | | | |  | Model 2 | | | |  | Model 3 | | | |
| --- | --- | --- | --- | --- | --- | --- | --- | --- | --- | --- | --- | --- | --- | --- |
|  | Exp (b) | *SE* | 95% CI | *p* |  | Exp (b) | *SE* | 95% CI | *p* |  | Exp (b) | *SE* | 95% CI | *p* |
| (Intercept) | 0.025 | 0.001 | [0.023, 0.028] | **< .001** |  | 0.025 | 0.001 | [0.023, 0.028] | **< .001** |  | 0.025 | 0.001 | [0.023, 0.028] | **< .001** |
| Gender (man) | 0.833 | 0.040 | [0.758, 0.915] | **< .001** |  | 0.837 | 0.040 | [0.762, 0.919] | **< .001** |  | 0.837 | 0.040 | [0.762, 0.920] | **< .001** |
| Gender (other) | 0.950 | 0.191 | [0.640, 1.409] | .798 |  | 0.950 | 0.191 | [0.641, 1.409] | .800 |  | 0.951 | 0.191 | [0.641, 1.410] | .802 |
| Loneliness | 0.871 | 0.078 | [0.730, 1.038] | .122 |  | 0.862 | 0.070 | [0.735, 1.010] | .067 |  | 0.857 | 0.067 | [0.736, 0.999] | **.048** |
| Positive individual life event | 0.951 | 0.040 | [0.876, 1.034] | .238 |  | 0.954 | 0.039 | [0.881, 1.034] | .252 |  | 0.956 | 0.039 | [0.883, 1.034] | .262 |
| Positive social life event | 1.058 | 0.035 | [0.992, 1.130] | .087 |  | 1.058 | 0.035 | [0.991, 1.128] | .089 |  | 1.058 | 0.035 | [0.991, 1.128] | .090 |
| Negative social life event | 1.052 | 0.018 | [1.017, 1.088] | **.003** |  | 1.051 | 0.018 | [1.017, 1.087] | **.004** |  | 1.050 | 0.017 | [1.017, 1.085] | **.003** |
| Positive episode | 1.899 | 0.100 | [1.713, 2.105] | **< .001** |  | 1.901 | 0.100 | [1.714, 2.107] | **< .001** |  | 1.900 | 0.100 | [1.714, 2.107] | **< .001** |
| Negative episode | 1.647 | 0.089 | [1.481, 1.832] | **< .001** |  | 1.648 | 0.089 | [1.482, 1.833] | **< .001** |  | 1.648 | 0.089 | [1.482, 1.833] | **< .001** |
| Depression | 0.971 | 0.077 | [0.831, 1.135] | .713 |  | 0.989 | 0.044 | [0.907, 1.079] | .808 |  |  |  |  |  |
| Loneliness × Positive episode | 0.932 | 0.114 | [0.732, 1.185] | .564 |  | 1.000 | 0.103 | [0.817, 1.224] | .997 |  | 1.000 | 0.103 | [0.817, 1.224] | .998 |
| Loneliness × Negative episode | 1.219 | 0.150 | [0.957, 1.551] | .109 |  | 1.196 | 0.131 | [0.965, 1.482] | .102 |  | 1.197 | 0.131 | [0.966, 1.483] | .100 |
| Positive individual life event × Positive episode | 0.939 | 0.054 | [0.839, 1.051] | .273 |  | 0.929 | 0.052 | [0.833, 1.037] | .190 |  | 0.929 | 0.052 | [0.833, 1.036] | .184 |
| Positive individual life event × Negative episode | 1.060 | 0.061 | [0.946, 1.187] | .314 |  | 1.065 | 0.059 | [0.955, 1.188] | .256 |  | 1.065 | 0.059 | [0.955, 1.188] | .259 |
| Positive social life event × Positive episode | 1.058 | 0.048 | [0.968, 1.156] | .212 |  | 1.061 | 0.048 | [0.972, 1.159] | .184 |  | 1.062 | 0.048 | [0.973, 1.159] | .180 |
| Positive social life event × Negative episode | 0.973 | 0.046 | [0.887, 1.067] | .565 |  | 0.972 | 0.046 | [0.887, 1.065] | .543 |  | 0.972 | 0.046 | [0.887, 1.066] | .546 |
| Positive episode × Depression | 1.126 | 0.126 | [0.904, 1.402] | .289 |  |  |  |  |  |  |  |  |  |  |
| Negative episode × Depression | 0.961 | 0.099 | [0.786, 1.176] | .702 |  |  |  |  |  |  |  |  |  |  |
| *N* | 1550 | | | |  | 1550 | | | |  | 1550 | | | |
| AIC | 7817.937 | | | |  | 7816.329 | | | |  | 7814.387 | | | |
| log-Likelihood | -3889.968 | | | |  | -3891.164 | | | |  | -3891.194 | | | |

*(Cont’d)*

|  | Model 4 | | | |  | Model 5 | | | |
| --- | --- | --- | --- | --- | --- | --- | --- | --- | --- |
|  | Exp (b) | *SE* | 95% CI | *p* |  | Exp (b) | *SE* | 95% CI | *p* |
| (Intercept) | 0.025 | 0.001 | [0.023, 0.028] | **< .001** |  | 0.025 | 0.001 | [0.023, 0.028] | **< .001** |
| Gender (man) | 0.839 | 0.040 | [0.764, 0.922] | **< .001** |  | 0.841 | 0.040 | [0.766, 0.924] | **< .001** |
| Gender (other) | 0.964 | 0.194 | [0.651, 1.429] | .856 |  | 0.974 | 0.196 | [0.657, 1.444] | .895 |
| Loneliness | 0.870 | 0.063 | [0.756, 1.002] | .054 |  | 0.873 | 0.060 | [0.764, 0.999] | **.048** |
| Positive individual life event | 0.951 | 0.037 | [0.881, 1.027] | .204 |  | 0.952 | 0.021 | [0.910, 0.995] | **.028** |
| Positive social life event | 1.072 | 0.021 | [1.031, 1.114] | **.001** |  | 1.074 | 0.021 | [1.033, 1.116] | **< .001** |
| Negative social life event | 1.051 | 0.017 | [1.017, 1.085] | **.003** |  | 1.048 | 0.017 | [1.014, 1.082] | **.005** |
| Positive episode | 1.908 | 0.100 | [1.721, 2.115] | **< .001** |  | 1.909 | 0.101 | [1.722, 2.117] | **< .001** |
| Negative episode | 1.649 | 0.090 | [1.482, 1.834] | **< .001** |  | 1.651 | 0.090 | [1.484, 1.836] | **< .001** |
| Depression |  |  |  |  |  |  |  |  |  |
| Loneliness × Positive episode | 0.939 | 0.085 | [0.786, 1.121] | .485 |  | 0.967 | 0.081 | [0.820, 1.141] | .692 |
| Loneliness × Negative episode | 1.240 | 0.117 | [1.031, 1.492] | **.022** |  | 1.202 | 0.106 | [1.012, 1.428] | **.036** |
| Positive individual life event × Positive episode | 0.952 | 0.050 | [0.858, 1.056] | .351 |  |  |  |  |  |
| Positive individual life event × Negative episode | 1.049 | 0.055 | [0.947, 1.161] | .362 |  |  |  |  |  |
| Positive social life event ×  Positive episode |  |  |  |  |  |  |  |  |  |
| Positive social life event × Negative episode |  |  |  |  |  |  |  |  |  |
| Positive episode × Depression |  |  |  |  |  |  |  |  |  |
| Negative episode × Depression |  |  |  |  |  |  |  |  |  |
| *N* | 1,550 | | | |  | 1,550 | | | |
| AIC | 7814.305 | | | |  | 7814.013 | | | |
| log-Likelihood | -3893.153 | | | |  | -3895.006 | | | |

*Note*. *N* = 1,550. *SE* = standard error, CI = confidence interval. The model used a negative binomial distribution with a log link and included the total number of words as an offset term. The estimates were exponentiated. Baseline categories were the neutral episode condition for episode and woman for gender. All continuous predictors were mean centered in each condition (group).

**Figure S1**

*Association Between Loneliness and the Use of Social Words in Dataset 4 with the* *Control Variables (N = 1,550)*


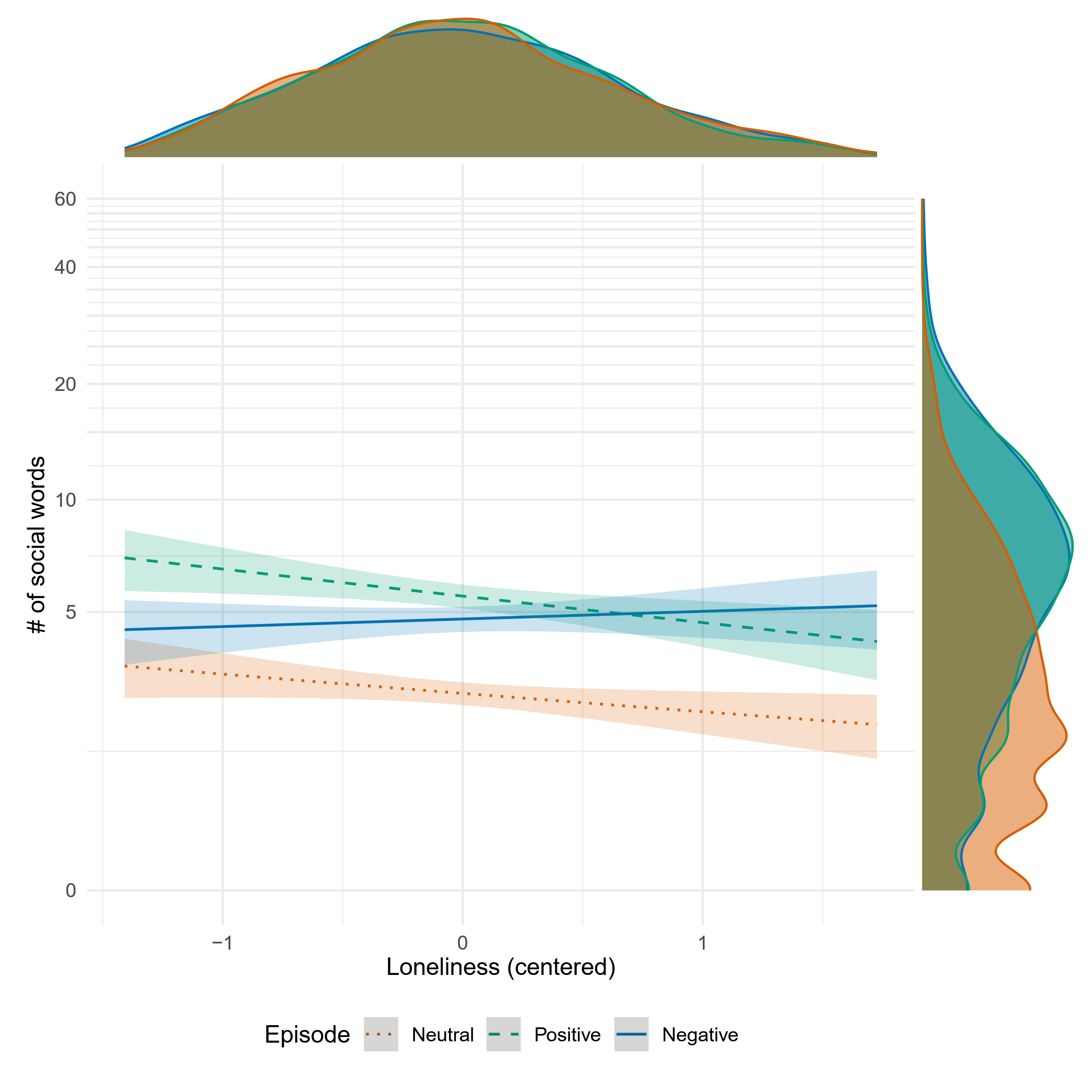


Note. Margins indicate 95% confidence intervals for the predicted values of each regressed line. The generalized linear mixed model (negative binomial distributions with log link) included the total number of words in each episode as an offset term and positive social life event, negative social life event, and positive individual life event as control variables (Model 5 in Table S6). Loneliness was mean centered in each condition.

# Reference

Iseki R. (2023). *anovakun* (Version 4.8.9) [A function running analysis of variance, simple effects, and multiple comparisons in R]. <https://riseki.cloudfree.jp/?ANOVA%E5%90%9B>
